# Experimental analysis and safety assessment of thermal runaway behavior in lithium iron phosphate batteries under mechanical abuse

**DOI:** 10.1038/s41598-024-58891-1

**Published:** 2024-04-15

**Authors:** Zhixiong Chai, Junqiu Li, Ziming Liu, Zhengnan Liu, Xin Jin

**Affiliations:** 1 Beijing Institute of Technology, National Engineering Research Center of Electric Vehicles, Beijing, 100081 China; 2 Beijing Institute of Space Launch Technology, Beijing, 100076 China

**Keywords:** Lithium–ion battery, Mechanical abuse, Internal short circuit, Thermal runaway, Safety assessment, Regression models, Electrical and electronic engineering, Mechanical engineering

## Abstract

Mechanical abuse can lead to internal short circuits and thermal runaway in lithium-ion batteries, causing severe harm. Therefore, this paper systematically investigates the thermal runaway behavior and safety assessment of lithium iron phosphate (LFP) batteries under mechanical abuse through experimental research. Mechanical abuse experiments are conducted under different conditions and battery state of charge (SOC), capturing force, voltage, and temperature responses during failure. Subsequently, characteristic parameters of thermal runaway behavior are extracted. Further, mechanical abuse conditions are quantified, and the relationship between experimental conditions and battery characteristic parameters is analyzed. Finally, regression models for battery safety boundaries and the degree of thermal runaway risk are established. The research results indicate that the extracted characteristic parameters effectively reflect internal short circuit (ISC) and thermal runaway behaviors, and the regression models provide a robust description of the battery's safety boundaries and thermal runaway risk degree. This work sheds light on understanding thermal runaway behavior and safety assessment methods for lithium-ion cells under mechanical abuse.

## Introduction

In the face of the dual challenges of the global energy crisis and environmental protection, developing new energy vehicles to replace traditional fossil fuel vehicles has gradually become a global consensus and trend^1^. As a core component of new energy vehicles, lithium-ion batteries have also experienced rapid development in recent years, and researchers carried out a large and systematic work from battery models^2–4^, battery thermal management systems (BTMS)^5–7^, and battery safety management^8–10^. However, ISC and thermal runaway caused by mechanical damage are still tricky and have constrained their development and application. Reports and statistics on fire/explosion incidents indicate that collisions are the leading cause of such incidents^11^.

Upon mechanical abuse such as collision, squeezing, and puncturing, batteries undergo deformation, damaging and rupturing internal components (separator, electrodes). The contact between the positive and negative electrodes causes ISC, generating a substantial amount of heat, subsequently resulting in thermal runaway, accompanied by phenomena such as leakage, smoke, and combustion^12–14^. Researchers have conducted extensive experiments, theoretical studies, and simulations in response to this issue. In terms of mechanical abuse experiments, researchers have explored experiments under quasi-static squeezing^15,16^, dynamic loads^17,18^, different shapes of punch^19,20^, different battery types^21–23^, and different battery SOC^17^. These experiments aim to capture changes in parameters such as voltage, force, and temperature during the battery's failure process. Techniques like electrochemical impedance spectroscopy (EIS)^24^, relaxation time distribution (DRT)^25^, and in-situ characterization methods are employed to analyze internal battery damage and observe changes in the microstructure of battery materials before and after damage^26^.

Addressing the issue of battery mechanical deformation due to squeezing, researchers, combining relevant structural mechanics theories, have developed models suitable for lithium-ion batteries, including the Ludwik isotropic hardening model^27^, Drucker-Prager model^28^, Johnson–Cook plastic model^16,29^. These models explain the stress–strain changes throughout the entire compression deformation process. Researchers apply material failure theories for the ISC issue resulting from mechanical squeezing, utilizing criteria such as the maximum principal stress criteria^15^, the Mohr–Coulomb criteria^15^, and unified strength criteria^17^, effectively predicting ISC problems.

Combining experimental and theoretical research, researchers, through simulation methods, have reproduced the coupled evolution behavior of mechanical–electrical-thermal multi-physical fields during the squeezing failure process of lithium-ion batteries^30–33^. Li established a homogenized model for pouch cells considering battery SOC and strain rate, accurately predicting force–displacement changes under different mechanical loading conditions^34^. Li proposed a structure-damage-based coupled model of mechanical–electrical-thermal to study the failure behavior of 18,650 lithium-ion batteries under mechanical abuse in the hard ISC stage^35^.

Furthermore, studies on battery safety assessment have been conducted in response to ISC and thermal runaway caused by mechanical abuse^36–39^. Based on mechanical abuse experiments, Ohneseit^40^ and Ellersdorfer^41^ conducted safety assessments by extracting characteristic parameters of battery failure for 18,650 batteries and pouch cells, respectively. Based on mechanical abuse experiments and models, Li proposed a data-driven method to predict the safety of batteries under different mechanical abuse conditions^42^.

However, it is worth noting that research on battery mechanical abuse primarily focuses on the pouch and 18,650 batteries, with limited studies on prismatic LFP batteries, and lacks systematic investigation. Additionally, the emphasis is placed on the battery failure point in experiments, theory, or simulation studies. Current research mainly explains battery material failure from the perspective of battery material failure, lacking studies that analyze it from the perspective of engineering applications based on abuse conditions. Lastly, there are limited safety assessments for battery mechanical abuse; some are still qualitative analyses. This study systematically investigated LFP batteries under mechanical abuse under different punches and SOC, extracting and analyzing characteristic parameters such as voltage, temperature, and force during the battery's failure process. Mechanical abuse conditions were quantified, and the relationship between abuse conditions and battery abuse behavior was analyzed. Regression models for battery safety assessment under mechanical abuse were constructed, including regression models for battery safety boundaries and the degree of thermal runaway risk. The remainder of the paper is organized as follows: Section "Materials and Methods" presents the experimental setup. Section “Results and discussions” introduces experimental results and characteristic parameter extraction under different abuse conditions. Section "Safety assessment of thermal runaway behavior" discusses the methods for battery mechanical abuse safety assessment. Finally, Section "Conclusions" states the main conclusions.

## Materials and methods

### 32Ah LFP battery

This paper uses a 32 Ah lithium iron phosphate square aluminum case battery as a research object. Table 1 shows the relevant specifications of the 32Ah LFP battery. The electrolyte is composed of a standard commercial electrolyte composition (LiPF_6_ dissolved in ethylene carbonate (EC):dimethyl carbonate (DMC):methyl ethyl carbonate (EMC): 2:3:5 in volume). Supplementary Fig. S1 online shows the three-dimensional battery geometry and computed tomography (CT) graphics containing two jell rolls.

**Table 1:** Specifications of 32Ah LFP battery.

Specifications	Values
Rated capacity	32 Ah
Rated voltage	3.2 V
Cutoff voltage range	2.5 V–3.65 V
Geometry	148 mm × 91.5 mm × 26.7 mm
Mass	725 ± 50 g
Cell type	Prismatic cell

### The experimental settings

A battery has been cycled three times, and the average value of three discharging capacity determines its capacity. A cycled procedure includes a "CC-CV" charge procedure, two rested procedures, and a CC discharge procedure. Supplementary Fig. S2 online presents the cycled procedure and CC-CV charging curves of the 32 Ah LFP Li-ion cell. To maintain the consistency of the batteries, batteries whose capacity deviates from the rated capacity by more than 5%.

Twenty batteries with no more than 5% consistency were selected and divided into five groups for the mechanical abuse experiment. Each battery group was adjusted to a different capacity(100%SOC, 80%SOC, 60%SOC, and 20%SOC).

**Figure 1:**
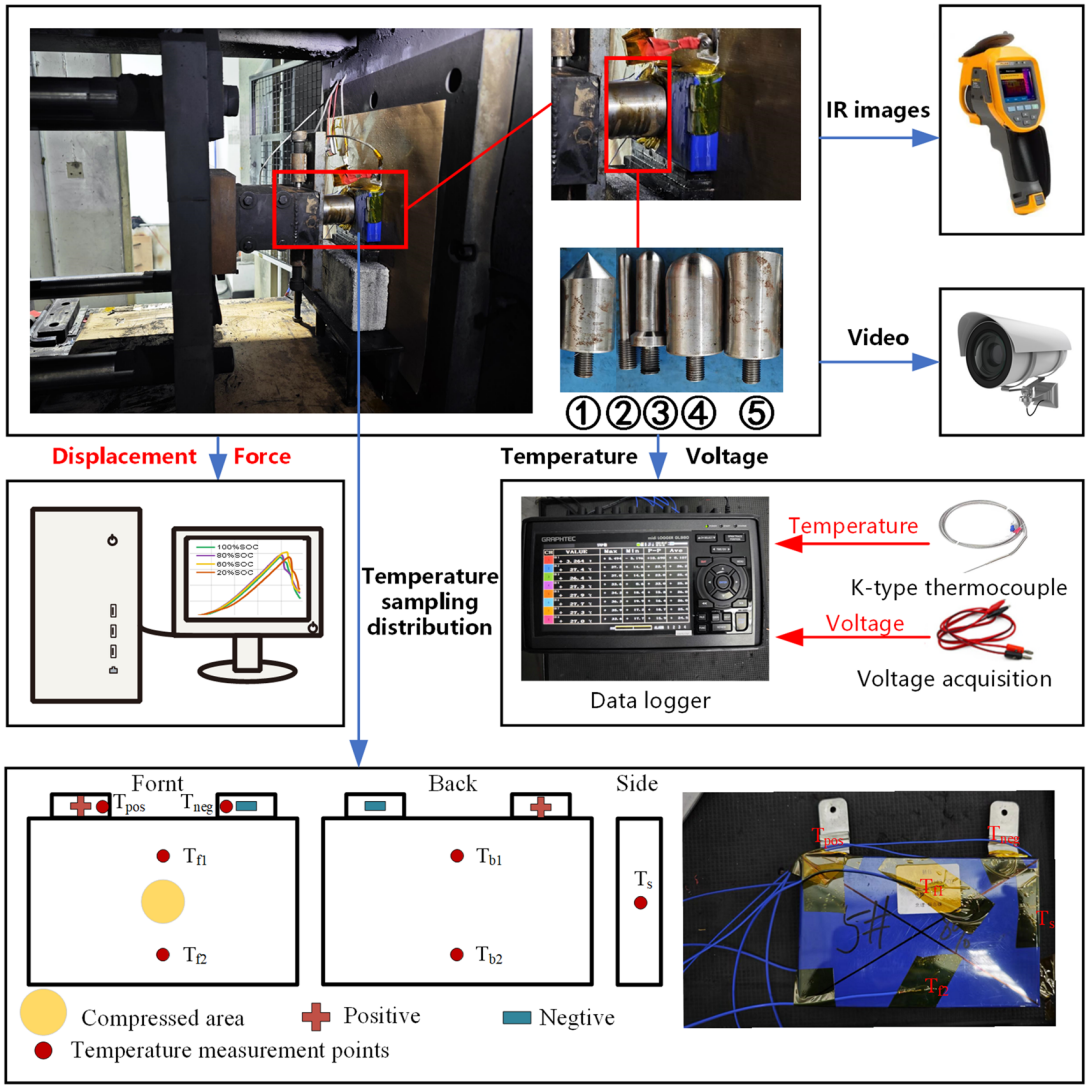
The schematic of the mechanical abuse experiment.

Five punches with different shapes and sizes were designed to simulate different mechanical abuse scenarios, as shown in Fig. 1. The punch sizes ranged up to 84% of the cell surface area, and the shapes included spherical, flat, and conical. Punches 2, 3, and 4 are the same shape but different sizes; punches 1, 4, and 5 are different but have the same largest diameter. The detailed punches loading experimental groupings are shown in Table 2.

**Table 2:** Experiment setup of abuse tests.

Group no	Battery no	Battery capacity	Punch sizes
1	1–4	100SOC, 80SOC, 60SOC, 20SOC	Conical 90°
2	5–8		Sphere ɸ15 mm
3	9–12		Sphere ɸ30 mm
4	13–16		Sphere ɸ60 mm
5	17–20		cylindrical plate ɸ60 mm

The schematic diagram of the test platform is depicted in Fig. 1. An RJD-ZJ pack nail penetration and crush tester with a maximum range of 200 kN, sourced from Shenzhen Ruijiada Technology Co., Ltd., was utilized to record displacement and pressure data during the experiments. A digital camera was employed to capture the dynamic changes in battery behavior during the experiments. A Ti401P infrared thermal imager, sourced from Fluke Corporation, was utilized to capture the thermal image of the battery. Seven K-type thermocouples, designated as T_pos_–T_s_ in Fig. 1, were affixed to the battery surface to record temperature data with an accuracy of ± 1°C and were connected to a data logger. The GL980 data acquisition instrument (DAQ), sourced from GRAPHTTEC Corporation, was used to collect battery temperature and voltage data at intervals of 100 ms.

Before testing, seven thermocouples were fixed on the surface of the Li-ion cell, connecting voltage acquisition, and then the cell was placed in the tester. The test speed was set at 30 mm/min. Maintain test environment at constant ambient temperature (25 ± 2 °C). During the test, the voltage, temperature, force, and displacement data are recorded in real time, a digital video camera captures images of the whole process, and a thermal imager records the temperature distribution of the battery after the thermal runaway.

To minimize experimental uncertainties, all instruments used in the experiments were calibrated before the start of the experiments and operated strictly according to the manufacturer's recommendations. Environmental conditions were maintained constant during the experiments, batteries were randomly assigned to experimental and control groups, and all factors that could affect the experimental results were recorded and controlled. This rigorous approach aimed to maximize the reduction of experimental errors. Additionally, three sets of repeat experiments ensured reproducibility before formal testing.

## Results and discussions

**Figure 2:**
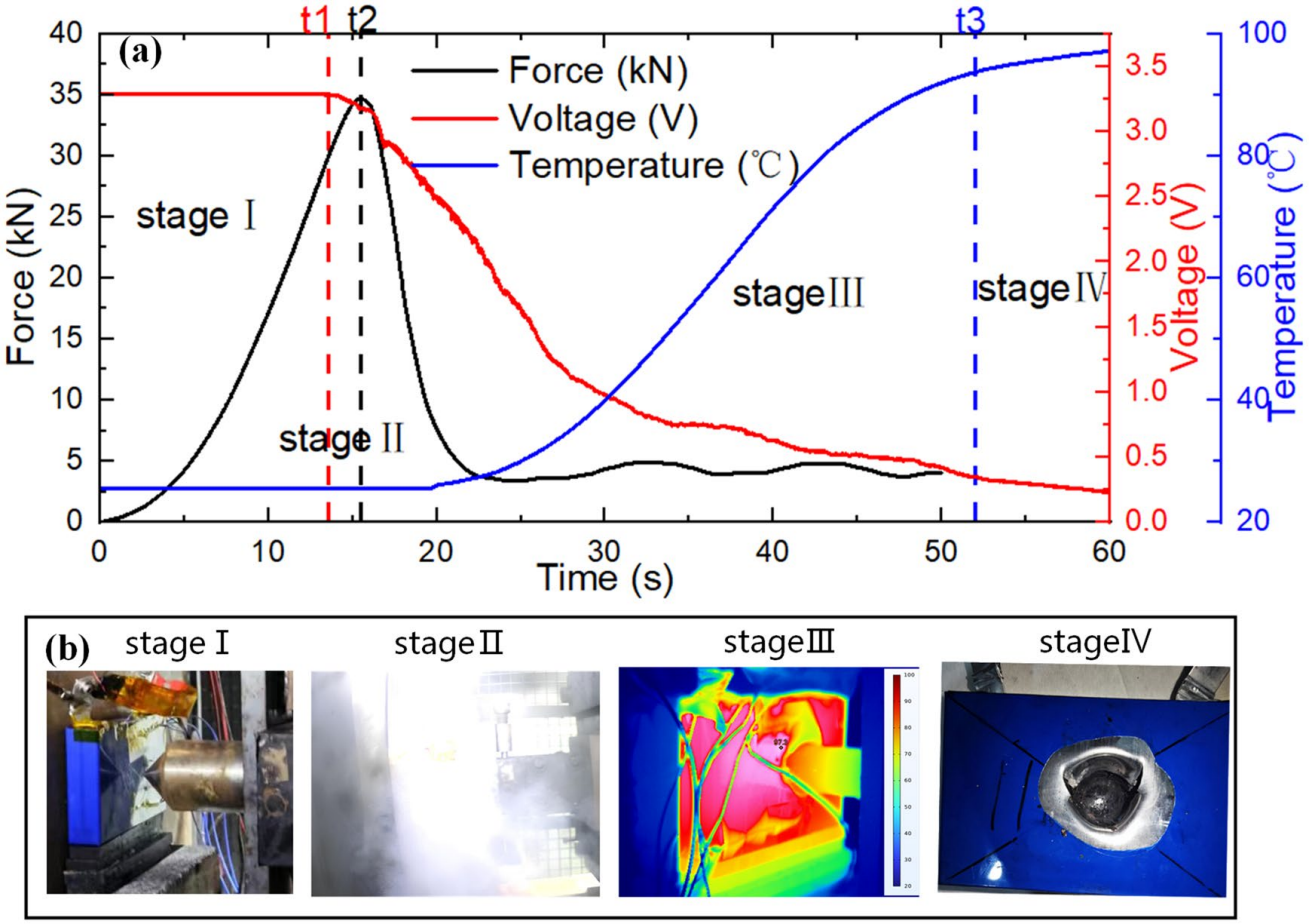
The experiment results of mechanical abuse (sphere ɸ30 mm, 60 SOC). (**a**) Mechanical–electrical-thermal response curve; (**b**) digital pictures and the infrared images of LFP cells during experiments.

In the case of a 60% SOC battery and a 30 mm hemispherical punch condition, Fig. 2 illustrates the mechanical–electrical-thermal response of the battery throughout the mechanical abuse process. The experimental process can be divided into four stages based on the changes in battery voltage, temperature, deformation, and force.

Stage I: Battery deformation stage. The battery gradually deforms as the punch intrudes, and the casing remains intact. At this stage, the force on the battery gradually increases with time/intrusion distance, and the voltage and temperature show no significant changes.

Stage II: Initiation of ISC. The battery voltage decreases with a voltage change rate ${{dV\left( t \right)} \mathord{\left/ {\vphantom {{dV\left( t \right)} {dt}}} \right. \kern-0pt} {dt}} > 10{{mV} \mathord{\left/ {\vphantom {{mV} s}} \right. \kern-0pt} s}$. ISC phenomena appear, emitting much white smoke, as shown in Fig. 2b. The force rises, while the temperature shows no significant change. Stage II is relatively short, lasting only 3.5 s in Fig. 2a.

Stage III: Thermal runaway stage. At this point, the force peaks, the battery casing ruptures, and the battery materials are damaged, causing a rapid force drop. As the punch intrudes into the battery, widespread ISC occurs, and the battery voltage rapidly drops to 0 V while the temperature rises rapidly, leading to thermal runaway.

Stage IV: Battery cooling stage. As the thermal runaway ends, the battery begins to cool slowly. In this case, except for the compressed and damaged areas, the battery still maintains a relatively intact appearance, but its internal materials undergo significant carbonization due to the thermal runaway reaction.

### Mechanical response and characteristic parameter extraction

**Figure 3:**
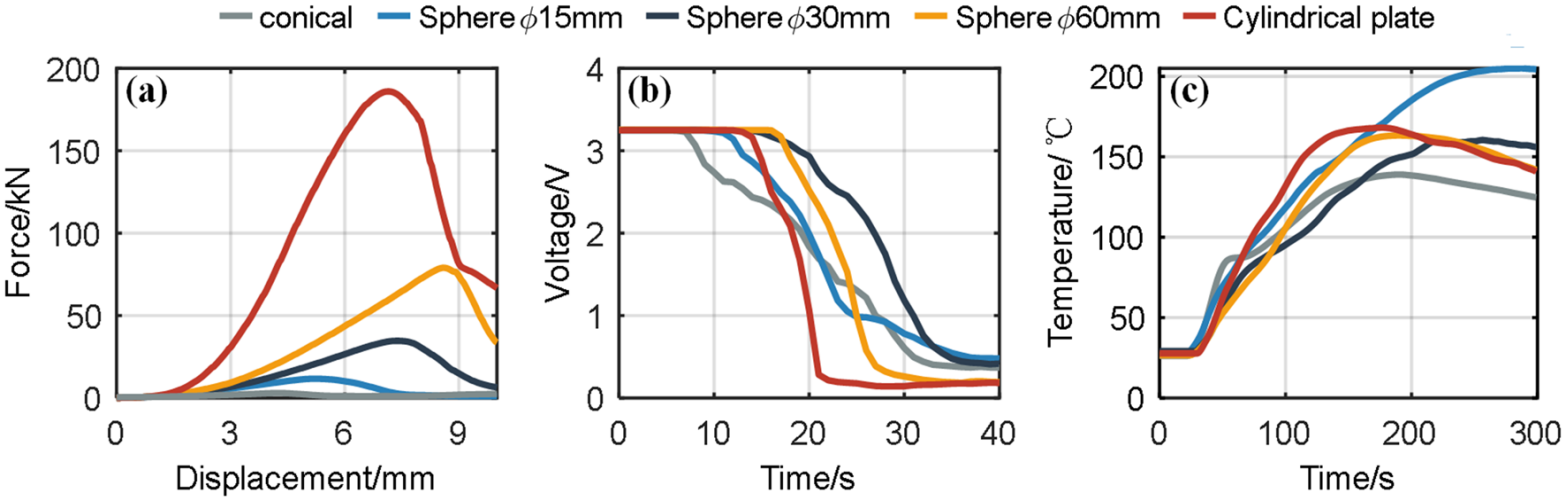
Comparison of response curves for 60% SOC batteries under mechanical abuse. (**a**) Pressure–displacement curve; (**b**) voltage–time curve; (**c**) temperature–time curve.

Supplementary Fig. S3 online shows the force–displacement data collected by the extrusion machine. The overall force–displacement curves for the battery are similar, with low stiffness in the early stage and a relatively smooth curve. In the later stage, the stiffness increases as the stacked electrodes in the battery gradually compact. Comparing the experimental results for different SOC batteries in each group, the force of different SOC batteries is similar, and the peak force and corresponding displacement are also similar. Therefore, the mechanical response of the LF32 battery is relatively independent of the battery SOC. Figure 3a takes 60% SOC batteries from each group, showing varied maximum force under different conditions, ranging from 3kN for the conical condition to 190kN for the cylindrical flat condition. Generally, the larger the force-bearing area of the battery, the greater the force it can withstand. The maximum force corresponds to different intrusion displacements, ranging from 3.59 mm for the conical condition to 8.63 mm for the 60 mm spherical punch condition. In general, battery failure can be explained from the perspective of material failure criteria. The battery separator material fails when subjected to particular stress or strain. A higher force is needed for a battery with a larger force-bearing area to achieve the same stress.

**Figure 4:**
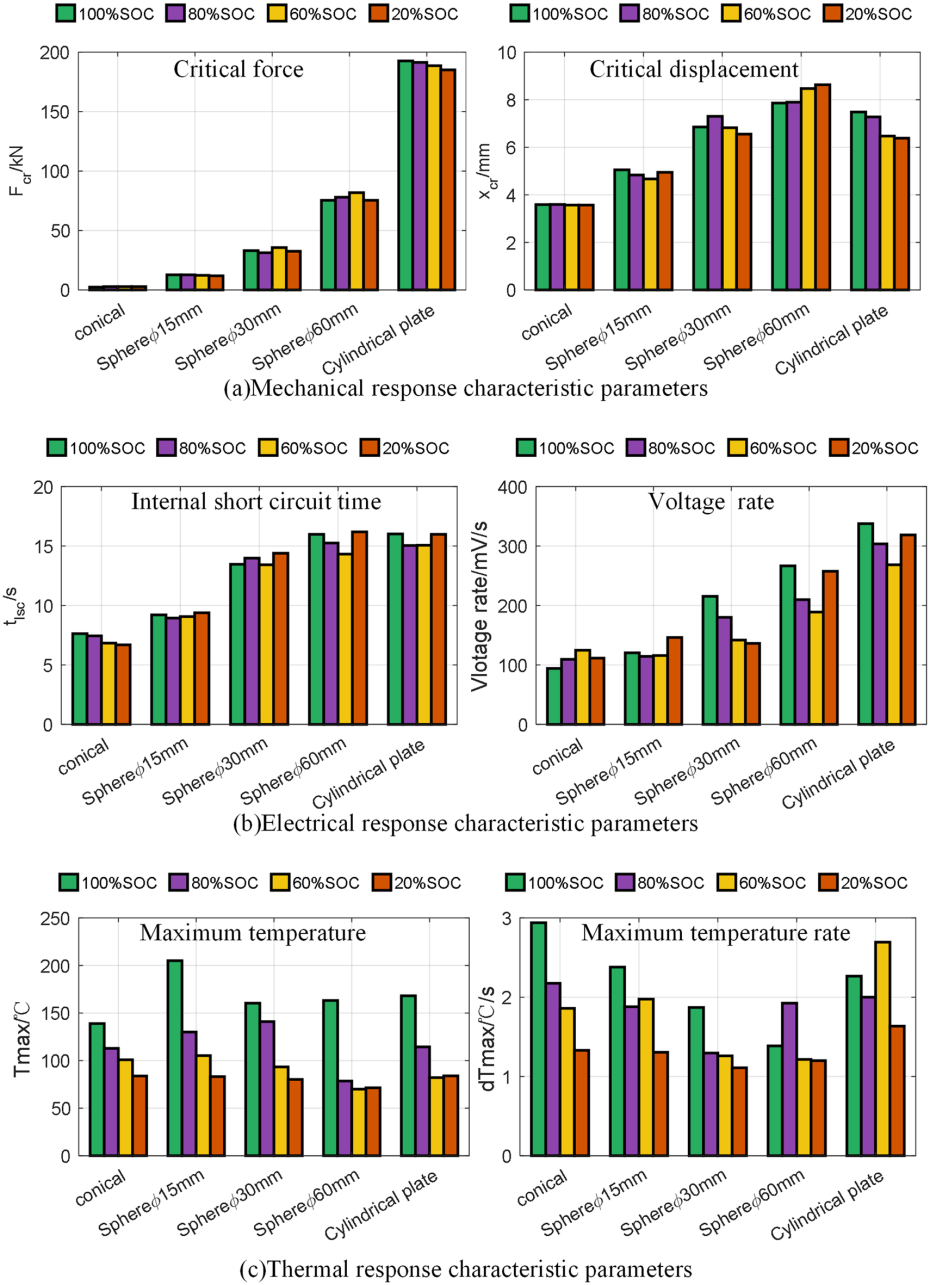
Comparison of battery characteristic parameters under mechanical abuse. (**a**) Mechanical response characteristic parameters; (**b**) electrical response characteristic parameters; (**c**) thermal response characteristic parameters.

To further compare the mechanical response of the LF32 battery, Eqs. (1) and (2) were used to extract the critical force $F_{cr}$ and the corresponding critical displacement $x_{cr}$ under different conditions, as shown in Fig. 4a. These two features reflect the critical state the battery can withstand before the casing breaks. Figure 4a shows that as the contact area between the punch and the battery increases, from conical to hemispherical to flat, the force the battery can withstand increases gradually. The overall trend of critical displacement for the battery is similar to critical force, only closer in numerical range. The maximum and minimum force difference is nearly 60 times, while the maximum and minimum displacement differs by less than 2.3 times.

1 $$F_{cr} = max(F(t))$$

2 $$x_{cr} = \left\{ {x_{t} \left| {\mathop {\arg max}\limits_{t} \left( {F(t)} \right)} \right.} \right\}$$

### Electrical response and characteristic parameter extraction

Comparing the experimental results for different SOC batteries in each group, the voltage decreases when Stage II starts, reaching below 1 V within about 20 s (Supplementary Fig. S4 online). The voltage–time curves for the battery are generally similar, and the electrical response of the LF32 battery is relatively independent of battery SOC. Figure 3b takes 60% SOC batteries from each group as an example, showing a similar overall decreasing trend. The moment of the ISC increases with the force-bearing area of the battery. The moment of the ISC is the smallest for the conical condition and largest for the plate condition. The voltage drop rate among different groups decreases with the force-bearing area of the battery. From the perspective of force, the larger the force-bearing area, the larger the area under force, and thus, the greater the force the battery can withstand. Therefore, when ISC occurs with a larger force-bearing area, the triggered failure area for ISC is more significant, resulting in a minor ISC resistance and a faster voltage drop rate for the battery.

Characteristic parameters of the electrical response under mechanical abuse were extracted: the moment of ISC and the voltage drop rate. The starting moment of the ISC $t_{isc}$ was determined when the voltage change rate reached ${{dV\left( t \right)} \mathord{\left/ {\vphantom {{dV\left( t \right)} {dt}}} \right. \kern-0pt} {dt}} > 10{{mV} \mathord{\left/ {\vphantom {{mV} s}} \right. \kern-0pt} s}$, as shown in the Eq. (3). Comparing the voltage drop curves, 1 V was chosen as the end time $t_{end}$ to comprehensively reflect the voltage drop rate. The Eq. (5) represents the voltage drop rate, reflecting the size of the internal failure area of the battery, with a more significant rate indicating a larger ISC failure area.

3 $$t_{isc} = \mathop {argmin}\limits_{t} (\left| {{{dV\left( t \right)} \mathord{\left/ {\vphantom {{dV\left( t \right)} {dt}}} \right. \kern-0pt} {dt}} - 0.01} \right|)$$

4 $$t_{end} = \mathop {argmin}\limits_{t} (\left| {V\left( t \right) - 1} \right|)$$

5 $$dV = \left| {\frac{{V_{end} - V_{isc} }}{{t_{end} - t_{isc} }}} \right|$$

Figure 4b shows $t_{isc}$ and $dV$ for the five groups of experiments. Comparing the experimental results $t_{isc}$, the deviation within the same group is within 7.2% and relatively independent of SOC. Between different groups, $t_{isc}$ increases with the force-bearing area of the battery. Comparing the experimental results $dV$, there is no clear relationship with SOC within the same group. Between different groups, $dV$ increases with the internal failure area of the battery.

### Thermal response and characteristic parameter extraction

The experiment equipped the battery surface with seven temperature sampling points. When the battery triggers ISC and enters Stage III, as the punch intrudes and thermal runaway occurs, the ISC area gradually extends from the front center point outward, eventually expanding to the entire battery (Supplementary Fig. S5 online). The heat generated by ISC and thermal runaway takes time to conduct within the battery, so the temperatures of T_ft_ and T_fl_ closest to the ISC area rise first, while the temperatures of the farthest battery terminals Tneg and Tpos rise more slowly. The battery's maximum temperature difference (MTD) is calculated according to the Eq. (6). The MTD quickly rises after the battery triggers ISC due to the rapid response of T_f1_ and T_ft_. As thermal runaway extends to the entire battery, MTD begins to decrease, and after 200 s, the temperature difference drops to 36 °C. To highlight the temperature response of the battery during ISC thermal runaway, T_ft_, representing the temperature with a faster response, was selected for further analysis.

6 $$MTD\left( t \right) = max\left( {\left| {T_{i} \left( t \right) - T_{j} \left( t \right)} \right|} \right)\;\;\;T_{i} ,T_{j} \in \left\{ {T_{pos} ,T_{neg} ,T_{ft} ,T_{fl} ,T_{bt} ,T_{bl} ,T_{s} } \right\}$$

Comparing the experimental results for different SOC batteries in each group, when Stage III starts, and the battery experiences ISC and thermal runaway, the temperature rapidly rises as thermal runaway extends from the central region to the entire battery (Supplementary Fig. S6 online). The temperature–time curves for the battery are generally similar, with high SOC batteries having higher energy, resulting in higher peak temperatures after the thermal runaway. Figure 3c takes 60% SOC batteries from each group as an example, showing that the highest temperature reached by the battery has no apparent correlation with the battery contact area.

Characteristic parameters of the thermal response under mechanical abuse were extracted: the maximum temperature of the battery and the maximum temperature rise rate, as shown in Eqs. (7) and (8). The maximum temperature of the battery is positively correlated with the enthalpy change^40^, usually representing the energy released by the battery during thermal runaway. Typically, 1 °C/s is a critical reference indicator for thermal runaway triggering. The larger the maximum temperature rise rate, the more intense the reaction during thermal runaway.

7 $$T_{max} = max\left( {T(t)} \right)$$

8 $$dT_{max} = max\left( {dT(t)/dt} \right)$$

Figure 4c plot $T_{max}$ and $dT_{max}$ for the battery. Comparing the results $T_{max}$ within the same group, the battery has less energy as SOC decreases, resulting in a minor enthalpy change corresponding to thermal runaway and, thus, a more minor $T_{max}$. Between different groups, there is no apparent pattern for $T_{max}$. The pattern for $dT_{max}$ is generally similar to $T_{max}$. Within the same group, $dT_{max}$ generally decreases with smaller SOC, while there is no clear pattern between different groups.

## Safety assessment of thermal runaway behavior

### Quantitative analysis of abuse conditions

This study starts by quantitatively analyzing the mechanical abuse conditions, considering variations in the punch's shape and the battery's SOC. Previous analyses indicate a correlation between the mechanical–electrical-thermal response of the battery and the compressed area of the battery. As the punch initially contacts the battery, the sharper the punch, the smaller the contact area (e.g., group 1). As the compression progresses, the overall diameter of the punch decreases, resulting in a smaller contact area (e.g., group 2). Therefore, this paper defines $H_{factor}$ and $d_{factor}$ to describe the sharpness of the punch and its overall diameter, respectively.

**Figure 5:**
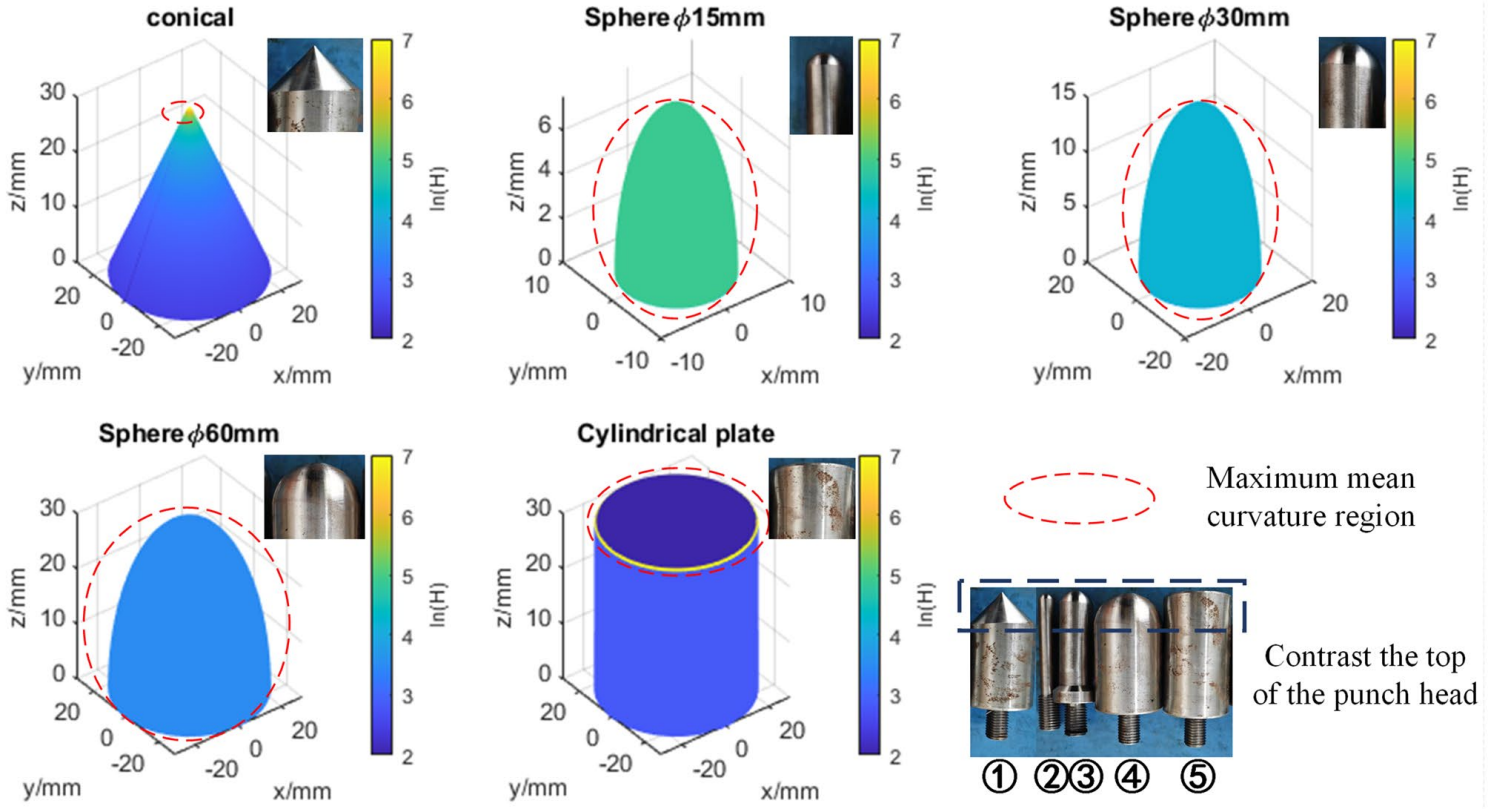
Three-dimensional geometry and mean curvature distribution of different punch.

To describe the sharpness of the punch, consider the mean curvature H, as shown in Eq. (9)^43^. Since the maximum mean curvature values in the five experiments are not in the same order of magnitude, take the logarithm of the maximum mean curvature as the descriptive quantity, as shown in the Eq. (10). The logarithmic distribution of the maximum mean curvature for the five experiments is illustrated in Fig. 5.

9 $$H = \frac{LG - 2MF + NE}{{2\left( {EG - F^{2} } \right)}}$$

10 $$H_{factor} = max\left( {\ln (H)} \right)$$

where $E$, $F$, $G$ are coefficients of the first fundamental forms coefficients of surface patch, and $L$, $M$, $N$ are coefficients of the second fundamental forms coefficients of surface patch.

The overall diameter of the battery is described by the three-dimensional diameter corresponding to the maximum curvature, as shown in the Eq. (11).

11 $$d_{factor} = \left\{ {d\left( {x,y,z} \right)\left| {\mathop {argmax}\limits_{x,y,z} \left( {H(x,y,z)} \right)} \right.} \right\}$$

In summary, $H_{factor}$ and $d_{factor}$ are used as two quantities to quantify the mechanical abuse conditions of the battery, as shown in Table 3. Moreover, battery SOC is used to quantify the battery's state.

**Table 3:** Different punch quantized analysis parameters.

Group no	Punch	Curvature factor	Diameter factor
Group1	Cone	6.92	1
Group2	Sphere ɸ15 mm	4.89	15
Group3	Sphere ɸ30 mm	4.20	30
Group4	Sphere ɸ60 mm	3.51	60
Group5	Cylindrical plate	6.97	60

### Thermal runaway safety boundary analysis

#### Safety boundary analysis based on experimental data

In the battery mechanical abuse experiment, although the battery voltage can react in advance to ISC, the actual operation of the battery often involves charging and discharging cycles, causing continuous voltage fluctuations. Therefore, small voltage fluctuations cannot be used to determine the battery safety boundary. Choosing mechanical characteristic parameters during battery mechanical abuse is a more reasonable approach. Considering that ISC slightly precedes the force peak, correction coefficients $\alpha_{F}$ and $\alpha_{x}$ are introduced, and in this experiment, $\alpha_{F} = 0.84$ and $\alpha_{x} = 0.88$ are calculated.

12 $$\left\{ {\begin{array}{*{20}c} {F_{safe} = \alpha_{F} F_{cr} } \\ {x_{safe} = \alpha_{x} x_{cr} } \\ \end{array} } \right.$$

**Figure 6:**
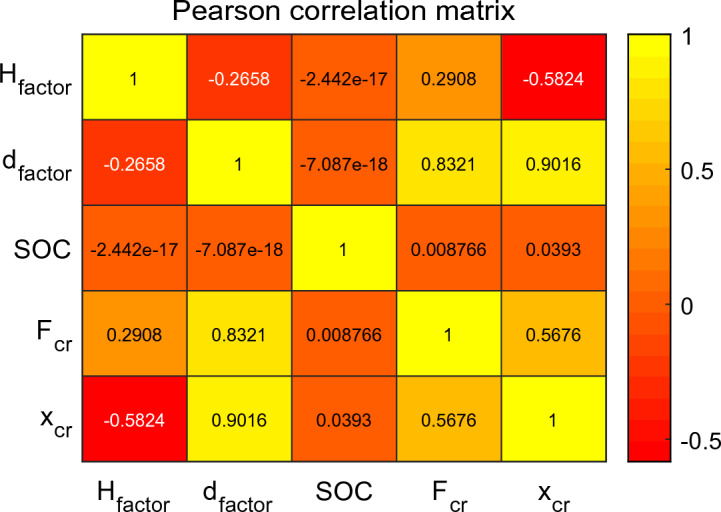
Pearson correlation coefficient analysis matrix: battery safety boundary correlation analysis.

This paper conducts Pearson linear correlation coefficient analysis for input variables (SOC, $H_{factor}$, $d_{factor}$) and output variables ($F_{cr}$ and $x_{cr}$), as shown in Fig. 6. Generally, correlation strength is judged based on the following ranges, as shown in Table 4. The absolute value of the correlation indicates the correlation's strength; the closer the coefficient is to 1 or − 1, the stronger the correlation, while the closer it is to 0, the weaker the correlation. According to the correlation coefficient analysis table, it can be observed from Fig. 6 that $F_{cr}$ is strongly correlated with $d_{factor}$, weakly correlated with $H_{factor}$, and not correlated with SOC; $x_{cr}$ is strongly correlated with $d_{factor}$, moderately correlated with $H_{factor}$, and not correlated with SOC.

**Table 4:** Correlation coefficient analysis table.

The absolute value of the correlation coefficient	Degree of correlation
0.8–1	Very strong correlation
0.6–0.8	Strong correlation
0.4–0.6	Middle correlation
0.2–0.4	Weak correlation
0.0–0.2	Very weak or no correlation

**Figure 7:**
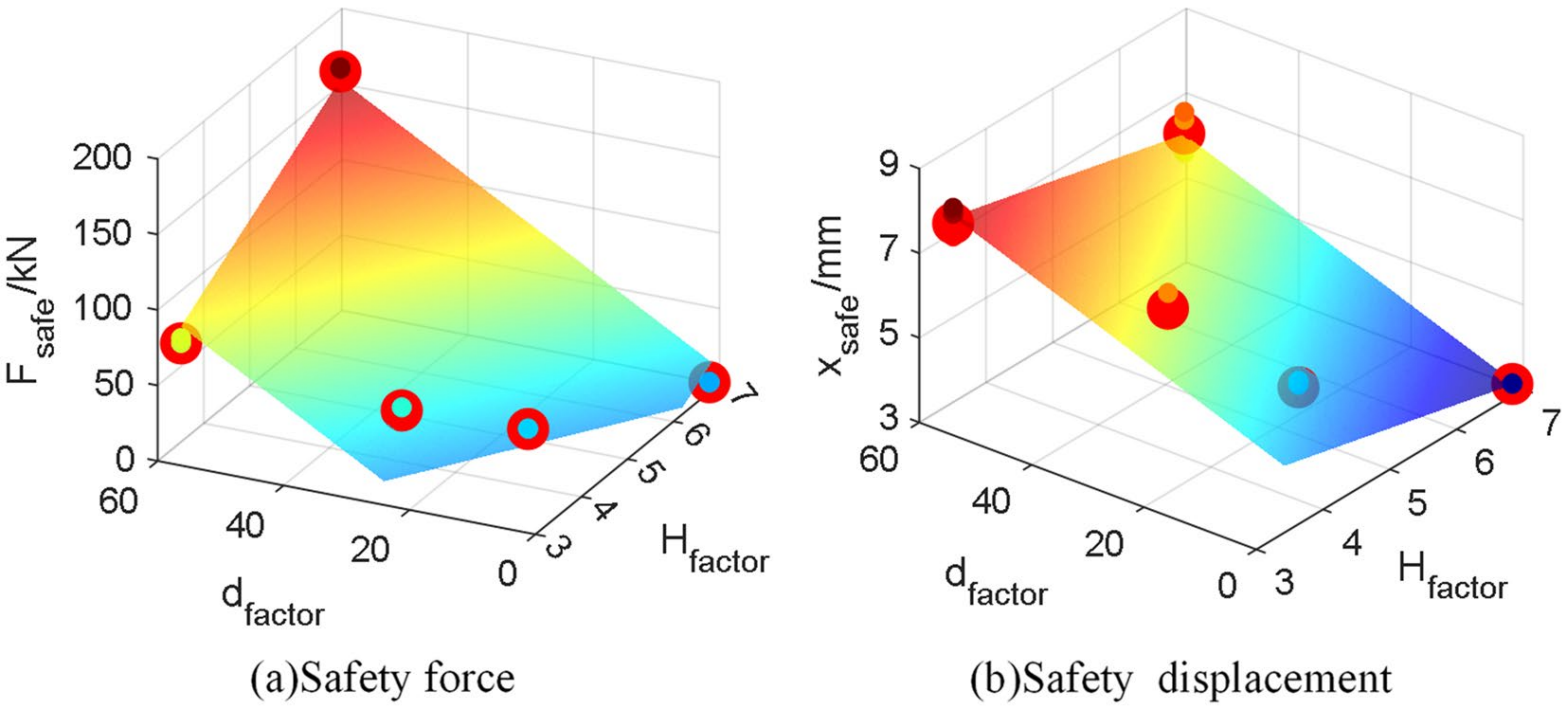
Battery safety threshold. (**a**) Force safety boundary; (**b**) displacement safety boundary.

Therefore, based on the above analysis, linear correlation function models for $F_{safe}$ and $x_{safe}$ with $H_{factor}$ and $d_{factor}$ are constructed, and parameters are estimated through the least squares method to obtain Eqs. (13) and (14). Considering that the regression model is multivariate, the adjusted $R^{2}$ is used to describe the fitting degree of the model. The adjusted $R^{2}$ is very close to 1, indicating a high fitting accuracy of the function model. Figure 7a,b respectively show the surfaces of $F_{safe}$ and $x_{safe}$, with black dots representing each experimental value and red dots representing the mean values of each abuse condition.

13 $$F_{safe} = \, \alpha_{F} \begin{array}{*{20}c} {\left( { - 171.3 + 26.6H_{factor} + 2.815{\text{d}}_{factor} } \right)} & {R^{2}_{adjusted} { = }0.9713} \\ \end{array}$$

14 $$x_{safe} = \alpha_{x} \left( {6.523 - 0.4368H_{factor} + 0.0567{\text{d}}_{factor} } \right) \, \begin{array}{*{20}c} {} & {R^{2}_{adjusted} { = }0.9322} \\ \end{array}$$

A significance test is conducted on the fitting parameters to validate the model and avoid overfitting. If the p-value is small, it indicates that the probability of the null hypothesis occurring is small, and if it occurs, according to the small probability principle, there is reason to reject the null hypothesis. In general, p > 0.05 indicates no significant difference; 0.01 < P < 0.05 indicates a significant difference; p < 0.01 indicates a highly significant difference. Table 5 shows the parameter values and corresponding p-values for the six model parameters, and the results indicate that all p-values are much less than 0.01. Therefore, the established linear regression model is highly reliable.

**Table 5:** Model significance test: P-value calculation.

Model	F_safe_		x_safe_	
	Parameter value	P value	Parameter value	P value
Constant	− 171.3	8.98E−11	6.523	7.63E−11
H_factor_	26.6	1.34E−10	− 0.4368	1.57E−05
d_factor_	2.815	1.24E−14	0.0567	3.03E−10

Based on the above analysis, the safety boundary of battery mechanical abuse strongly linearly correlated with $H_{factor}$ and $d_{factor}$ is obtained. The safety force $F_{safe}$ is positively correlated with $H_{factor}$ and $d_{factor}$, and the displacement boundary $x_{safe}$ is negatively correlated with $H_{factor}$ and positively correlated with $d_{factor}$. The linear regression model established for the safety boundary allows for a convenient and efficient determination of the battery's safety boundary based on operating conditions, aiding practical engineering applications.

#### Safety boundary analysis based on finite element model

However, the above analysis only provides a representation of the safety boundary, and further explanation of its inherent correlation needs to be explained through mechanisms. In current research, the safety boundary of the battery can be described through failure criteria, commonly divided into Stress-based failure criteria and Strain-based failure criteria^44,45^. These criteria are usually analyzed by calculating the internal stress of the battery. When calculating the strain of the battery, it is necessary to know the magnitude of the force on the battery and the area of the force cross-section. In the experimental situation, the force on the battery can be obtained through the extrusion machine, but the force cross-sectional area is challenging to obtain in real time. Considering that the square battery's metal shell undergoes deformation similar to a beam structure under compression, making it challenging to obtain the force cross-sectional area through analytical methods, a finite element method is adopted to calculate the internal stress of the battery.

**Figure 8:**
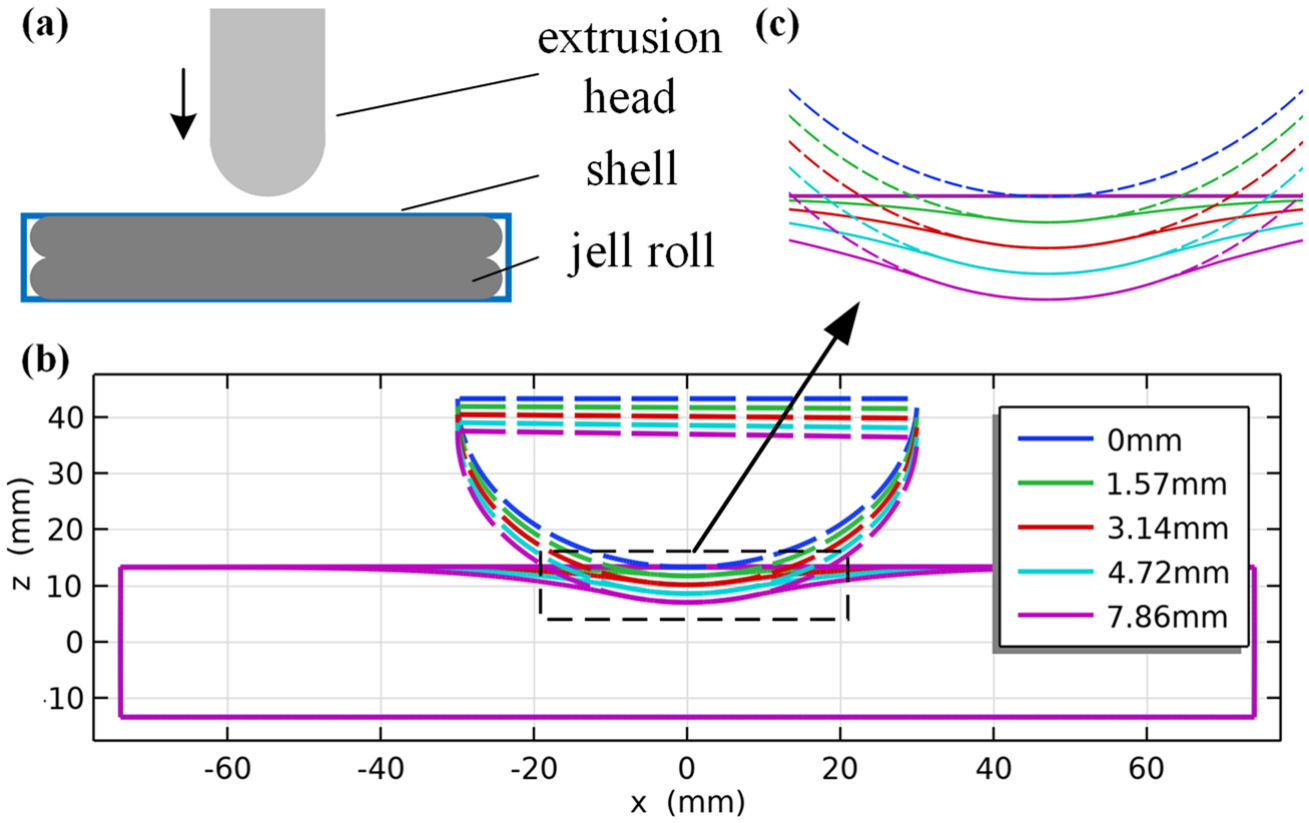
Finite element modeling diagram. (**a**) Geometric diagram of extrusion process; (**b**) extrusion process battery and punch section; (**c**) enlarged diagram of battery force section.

Figure 8a shows the finite element modeling schematic geometry, including the compression punch, battery shell, and two battery cores. Taking the 60 mm spherical punch as an example, Fig. 8b shows the schematic of the battery's deformation section with the punch's intrusion during the experiment. Zooming in on the contact surface between the battery and the punch, as shown in Fig. 8c, significant deformation occurs in the part of the battery shell that is not directly in contact with the punch after being pressurized. The finite element method can more conveniently simulate this scenario than analytical methods.

The battery shell and the punch are made of structural steel, and their modulus, density, and other parameters can be obtained by consulting literature. The battery core must undergo a modulus testing experiment to obtain its stress–strain curve. The stress–strain curve of the battery core was obtained from the flat plate extrusion experiment fitted using the isotropic model^46^.

15 $$\sigma_{p} = A\varepsilon_{p}^{n} + B$$

where A and B are parameters obtained through experiments, $\sigma_{p}$ is plastic stress, $\varepsilon_{p}^{{}}$ is plastic strain, and n is the hardening index.

Reference^47^ considers separator rupture when the internal stress of the battery reaches 43 MPa, which is the critical point for ISC in the battery. In this study, von Mises failure criteria $\sigma_{e} = 40MPa$ was adopted as the stress failure condition of the LF32 battery. The von Mises stress calculation is shown in the Eq. (16), where $\sigma_{1}$, $\sigma_{2}$, and $\sigma_{3}$ are the principal stresses of the material^48^.

16 $$\sigma_{e} = \sqrt {\frac{{\left( {\sigma_{1} - \sigma_{2} } \right)^{2} + \left( {\sigma_{2} - \sigma_{3} } \right)^{2} + \left( {\sigma_{3} - \sigma_{1} } \right)^{2} }}{2}}$$

Table 6 presents the parameters of the model. The model was simulated using COMSOL software to calculate the stress distribution of the battery under different mechanical abuses and the corresponding failure areas. The simulation boundary conditions were set: punch intrusion speed of 30 mm/min, intrusion distance combined with experimental results, and different punches corresponding to ISC displacement.

**Table 6:** Model parameter table.

Parameter	Value	Unit	Parameter	Value	Unit
Young's modulus (battery)^a^	300	MPa	B^b^	0.8	MPa
Poisson's ratio (battery)^46^	0.15	1	Young's modulus (punch)^c^	200	GPa
Density (battery)^a^	2000	kg/m^3^	Poisson's ratio (punch)^c^	0.3	1
Hardening index^b^	2.7		Density (punch)^c^	7850	kg/m^3^
A^b^	8135	MPa	Friction coefficient^27^	0.3	1

^a^Experimental measurement results.

^b^Parameter fitting results.

^c^Reference from the software database.

**Figure 9:**
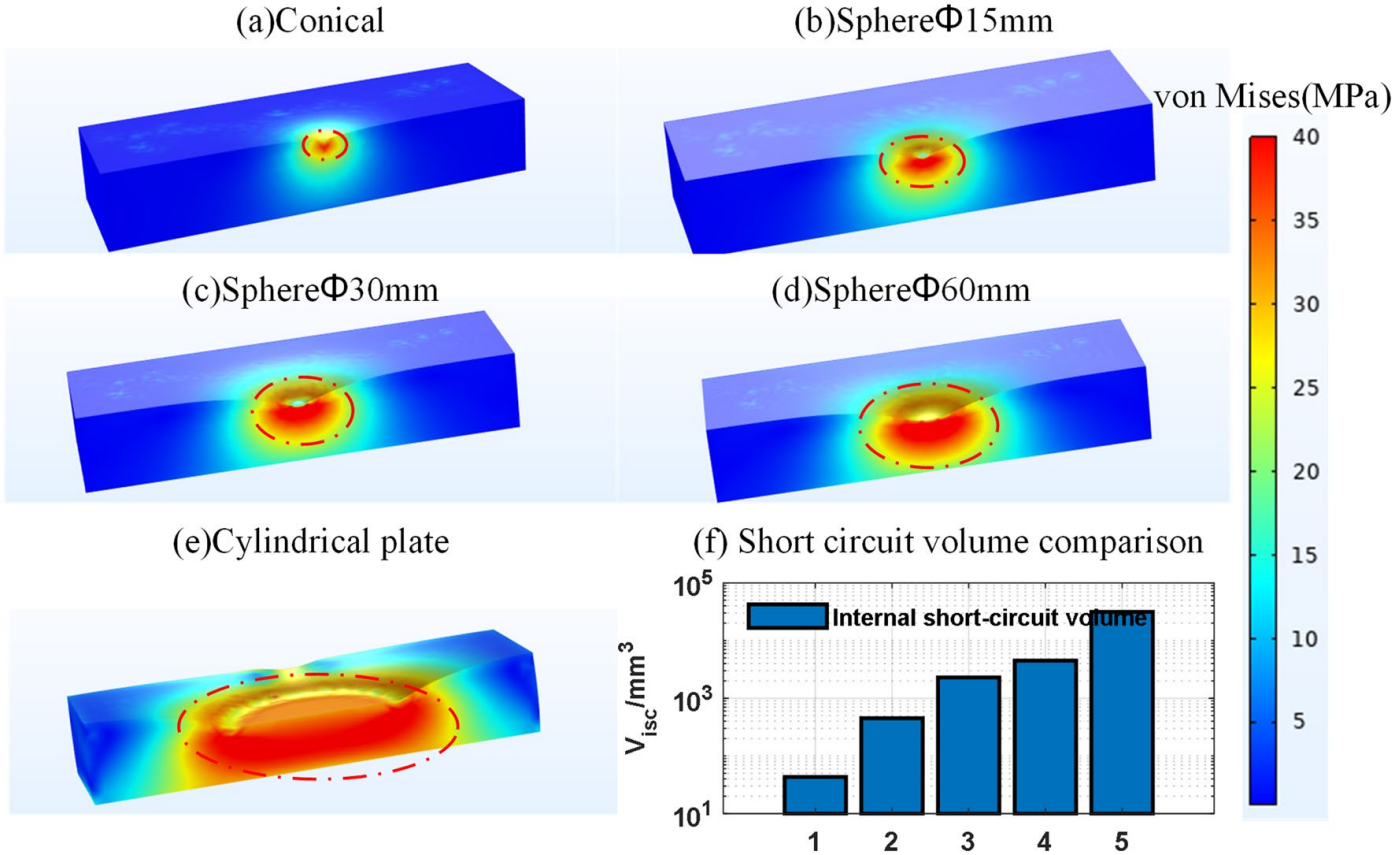
Critical state finite element simulation results. (**a**–**e**) Battery stress distribution in a critical state with different groups; (**f**) volume comparison of ISC region in different groups.

The simulation results of this study are shown in Fig. 9a–e, which plots the displacement and stress distribution of the battery in the corresponding experiments with 40 MPa as the stress upper bound. Comparing the simulation results of different battery groups, the larger the deformation, the higher the stress. Taking 40 MPa as the ISC boundary, the larger the compressed area of the battery, the larger the ISC area, as shown in Fig. 9f. Therefore, although the critical displacement and force for failure of the battery vary among different groups, the internal stress distribution pattern is relatively consistent and consistent with experimental analysis.

### Thermal runaway risk degree analysis

Upon exceeding the safety boundary, the battery will trigger the thermal runaway, leading to dangerous situations such as smoke and fire. The degree of thermal runaway risk is defined by calculating the total heat released during the entire thermal runaway process of the battery. Considering that the temperature difference of the battery has decreased after 200 s of the experiment, an analysis of the thermal images at 200 s is conducted. It is observed that, in addition to the temperature rise of the battery, the thermal runaway of the battery also causes a temperature increase in the punch and the steel block at the bottom of the battery. Thermal images of two typical experiments are shown in Supplementary Fig. S8 online.

The convection heat dissipation with less influence within 200 s is ignored in the calculation. The enthalpy change during the experimental process is calculated using the Eq. (17)

17 $$\Delta H = \sum\limits_{i = battery,punch,block} {M_{i} Cp_{i} (T_{i} - T_{i\_0} )}$$

where $i = battery,punch,block$ represents the battery, punch, and bottom block; $M_{i}$ is the mass; $Cp_{i}$ is the specific heat capacity; $T_{i}$ is the average temperature at 200 s; and $T_{i\_0}$ is the initial temperature. Relevant parameters are shown in Table 7.

**Table 7:** Enthalpy change calculation related parameters table.

	M/kg	Cp/J/(kg K)	T/°C	T0/°C
Battery	0.74	1017	a	25
Punch	b	475	a	25
Block	1.884	475	a	25

^a^Experimental data acquisition.

^b^Calculation based on different punch shapes.

**Figure 10:**
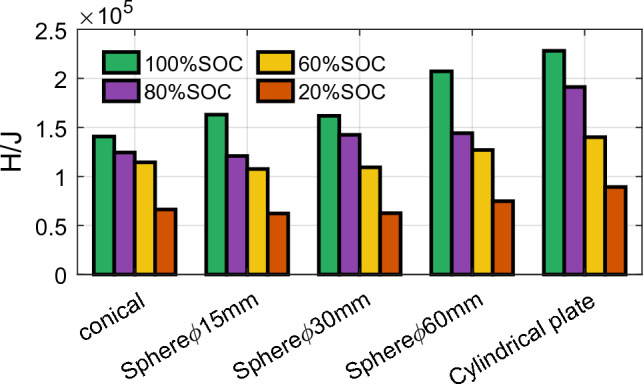
Enthalpy change under different SOC with different punches.

Figure 10 presents each condition's enthalpy change results for different SOC batteries. Within the same group, batteries with higher SOC release more energy during thermal runaway, and the enthalpy change increases with increasing SOC. Among different groups, the larger the compressed area of the battery, the higher the degree of ISC, resulting in a more significant heat release during ISC. The enthalpy change increases with the increase in the compressed area of the battery. This analysis result is consistent with the analysis result of the battery ISC in the electrical response in “Results and discussions”.

To define the risk degree of the battery, an analysis of the battery's post-experimental state is needed to establish a baseline. The appearance of 20% SOC and 60% SOC batteries remains relatively intact except for the compressed area, while 80% SOC and 100% SOC batteries exhibit prominent burn areas outside the compressed region, as shown in Supplementary Fig. S9 online.

Batteries with SOC below 60% have a relatively small impact on the surroundings after thermal runaway. Therefore, conical 60% SOC is selected as the baseline, and normalization is applied. The thermal runaway risk degree $Severty$ is defined and $Severty$ is related to the experimental group and the battery SOC, as shown in Eq. (18).

18 $$Severty\left( {i,SOC} \right) = \frac{{\Delta H\left( {i,SOC} \right)}}{{\Delta H\left( {1,60} \right)}} \, \begin{array}{*{20}c} {gruop} & {number} \\ \end{array} i,i = 1,2,3,4,5$$

A theoretical analysis of the thermal runaway during battery mechanical abuse is conducted to further analyze the relationship between the risk degree of batteries in the same group and different groups. Enthalpy change during mechanical abuse mainly includes heat generation from ISC ($Q_{isc}$) and side reactions in the battery ($Q_{side}$), as shown in the Eq. (19).

19 $$\Delta H = Q_{side} + Q_{isc}$$

**Figure 11:**
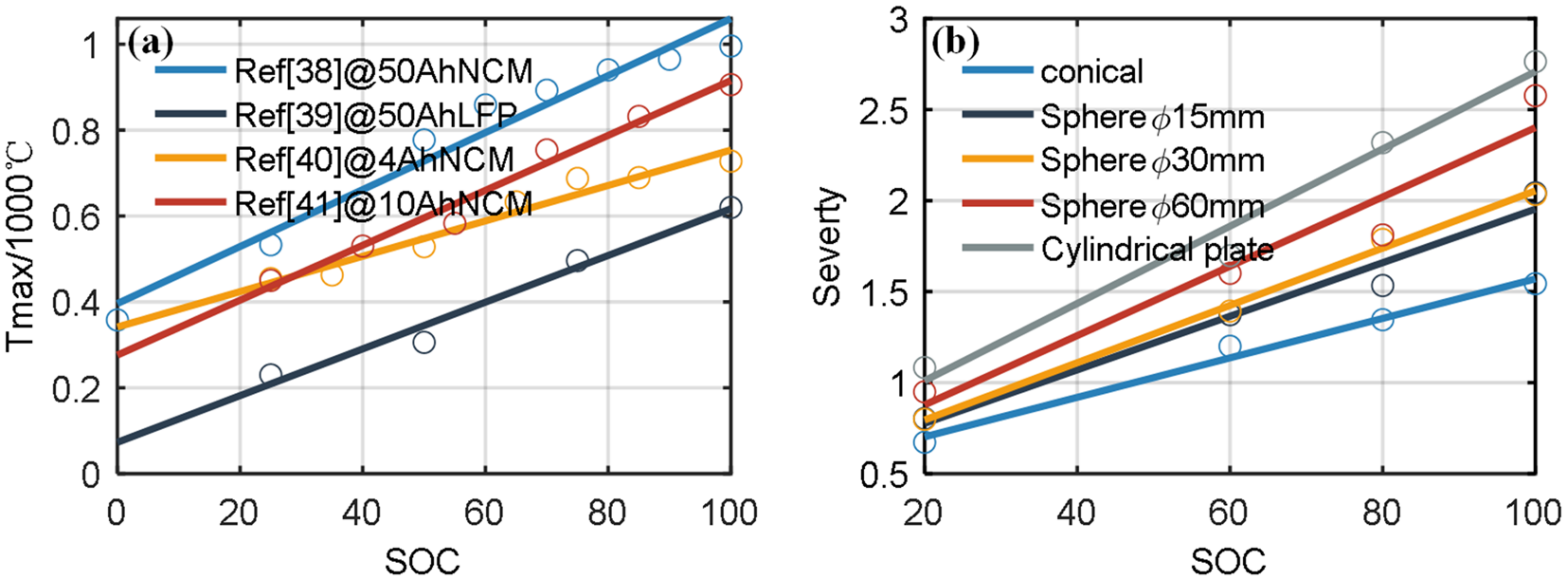
(**a**) The relationship between SOC and thermal runaway maximum temperature in the literature; (**b**) the relationship between SOC and risk degree of different battery groups in the experiment.

For $Q_{side}$, experiments in literature^49–52^ have conducted ARC thermal runaway experiments, and the Eq. (20) shows the relationship between the battery's highest temperature $T_{max}$ and $Q_{side}$. Experimental data show that the highest temperature of the battery is proportional to the battery's SOC, as shown in Fig. 11a, and all $R^{2}$ values were above 0.954.

20 $$T_{max} - T_{0} = \frac{{Q_{side} }}{{MC_{p} }} = a_{ref} SOC + b_{ref}$$

For $Q_{isc}$, within the same group, the punch is the same, and the battery is damaged similarly, with similar voltage drop rates and degrees of ISC^35^, so $Q_{isc}$ is almost the same. For different groups, $Q_{isc}$ is calculated using the Eq. (21). $V(t)$, $I_{isc} (t)$, and $r_{isc} (t)$ are difficult to obtain from experiments directly and usually require further combined accurate mechanical–electrical-thermal coupling models for simulation analysis^47,53–56^.

21 $$Q_{isc} = \int {V(t)I_{isc} (t)dt} = \int {\frac{{V(t)^{2} }}{{r_{isc} (t)}}dt}$$

Therefore, due to similar $Q_{isc}$ and $Q_{side}$ linearly correlated with SOC within the same group, combining Eqs. (18) and (19) a linear relationship between battery SOC and risk degree can be established, as shown in the Eq. (22). $Q_{isc}$ cannot be directly calculated for different groups, making quantitative analysis challenging.

22 $$Severty_{i} \left( {SOC} \right) = a_{i} {\text{SOC + b}}_{i} \, \begin{array}{*{20}c} {gruop} & {number} \\ \end{array} i,i = 1,2,3,4,5$$

Figure 11b shows the risk degree of batteries with different SOC in five experimental groups. The risk degree is strongly linearly related to the battery's SOC, the value of $R^{2}$ all above 0.936. In different groups, the risk degree of the battery increases with the increase in the area of the battery failure region but does not show a strong linear relationship. The Pearson correlation coefficients of $Severty$ with $d_{factor}$ and $H_{factor}$ are 0.37 and 0.08, indicating a relatively low linear correlation degree.

## Conclusions

The proposed experimental study provides a deep insight into battery thermal runaway behavior and safety assessment under mechanical abuse. The main conclusions are as follows:

1. Mechanical abuse experiments: Experiments under different punch shapes and state of charge (SOC) conditions revealed that the punch influences the pressure, voltage, and temperature of the battery during failure. Smaller punches result in lower pressures and displacements during battery failure, and a higher SOC results in a more severe thermal runaway.
2. Feature parameter extraction: Key parameters such as maximum pressure, voltage drop rate, and highest temperature were extracted from battery response curves.
3. Safety boundary analysis: $H_{factor}$ and $d_{factor}$ indices were introduced to quantify the mechanical abuse conditions, and a regression model for the battery's safety boundary was established with high accuracy.
4. Thermal runaway risk assessment: The system enthalpy change during battery thermal runaway was analyzed, and a regression model for thermal runaway risk was proposed. The risk of thermal runaway in batteries was found to be correlated with SOC and the size of the failure area.

In summary, this study systematically investigated the performance of LFP batteries under mechanical abuse and established corresponding models for safety boundaries and thermal runaway risk assessment.

### Supplementary Information

Supplementary Information.

## Data Availability

The data that support the findings of this study are available on request from the corresponding author.

## Abbreviations

BTMS: Battery thermal management systems
C-rate: The current rate, representing the charge/discharge current rate with respect to its nominal capacity
CC: Constant current charge/discharge procedure
CC-CV: Constant current–constant voltage charge procedure
CT: Computed tomography
CV: Constant voltage charge procedure
DAQ: Data acquisition instrument
DMC: Dimethyl carbonate
DRT: Relaxation time distribution
EC: Ethylene carbonate
EIS: Electrochemical impedance spectroscopy
EMC: Methyl ethyl carbonate
ISC: Internal short circuit
LFP: Lithium iron phosphate
MTD: Maximum temperature difference (°C)
SOC: State of charge (%)

## Variables

A/B: The coefficients of the stress–strain curve
$Cp_{i}$: The specific heat capacity of material I (J/(kg K))
$d_{factor}$: The diameter factor of the punch
$d(x,y,z)$: The overall diameter curvature at a point in space (x,y,z)
$dT_{max}$: The maximum temperature rate (°C/s)
$dV$: The voltage drop rate (mV/s)
${{dV(t)} \mathord{\left/ {\vphantom {{dV(t)} {dt}}} \right. \kern-0pt} {dt}}$: The voltage rate with respect to experiment time t (mV/s)
${{{E \mathord{\left/ {\vphantom {E F}} \right. \kern-0pt} F}} \mathord{\left/ {\vphantom {{{E \mathord{\left/ {\vphantom {E F}} \right. \kern-0pt} F}} G}} \right. \kern-0pt} G}$: Coefficients of the first fundamental forms coefficients of surface patch
$F_{cr}$: The critical force of mechanical response (kN)
$F_{safe}$: The force safety boundary
$F(t)$: The force with respect to experiment time t (kN)
$H$: The mean curvature
$H_{factor}$: The average curvature factor of punch
$H(x,y,z)$: The mean curvature at a point in space (x,y,z)
$I_{isc} (t)$: The ISC current with respect to experiment time t (A)
${{{L \mathord{\left/ {\vphantom {L M}} \right. \kern-0pt} M}} \mathord{\left/ {\vphantom {{{L \mathord{\left/ {\vphantom {L M}} \right. \kern-0pt} M}} N}} \right. \kern-0pt} N}$: Coefficients of the second fundamental forms coefficients of surface patch
$M_{i}$: The mass of material I (kg)
n: The hardening index of the stress–strain curve
$Q_{isc}$: The heat generation from ISC (J)
$Q_{side}$: The heat generation from side reactions (J)
$R^{2}_{adjusted}$: The adjusted coefficient of determination
$r_{isc} (t)$: The ISC resistance with respect to experiment time t (Ω)
$Severty$: The thermal runaway risk degree
${{T_{bt} } \mathord{\left/ {\vphantom {{T_{bt} } {T_{bl} }}} \right. \kern-0pt} {T_{bl} }}$: Temperature of the top/low of the back of the battery (°C)
${{T_{ft} } \mathord{\left/ {\vphantom {{T_{ft} } {T_{fl} }}} \right. \kern-0pt} {T_{fl} }}$: The temperature of the top/low of the front of the battery (°C)
$T_{i}$: The average temperature at 200 s of material i (°C)
$T_{i\_0}$: The initial temperature of material i (°C)
$T_{i} (t)/T_{j} (t)$: The temperature at measuring point i/j (°C)
$T_{max}$: The maximum temperature (°C)
${{T_{neg} } \mathord{\left/ {\vphantom {{T_{neg} } {T_{pos} }}} \right. \kern-0pt} {T_{pos} }}$: Battery negative/positive temperature (°C)
$t_{end}$: The moment the voltage drops to 1 V (s)
$t_{isc}$: The start moment of the ISC (s)
$V_{end}$: The voltage of the battery with respect to experiment time $t_{end}$ (V)
$V_{isc}$.: The voltage of the battery with respect to experiment time $t_{isc}$ (V)
$V(t)$: The voltage with respect to experiment time t (V)
$x_{cr}$: The critical displacement of mechanical response (mm)
$x_{safe}$: The displacement safety boundary
$x_{t}$: The displacement with respect to experiment time t (mm)

## Greek symbols

$\alpha_{x}$: The correction coefficients of the displacement safety boundary
$\alpha_{F}$: The correction coefficients of the force safety boundary
$\varepsilon_{p}$: The plastic strain
${{{{\sigma_{1} } \mathord{\left/ {\vphantom {{\sigma_{1} } {\sigma_{2} }}} \right. \kern-0pt} {\sigma_{2} }}} \mathord{\left/ {\vphantom {{{{\sigma_{1} } \mathord{\left/ {\vphantom {{\sigma_{1} } {\sigma_{2} }}} \right. \kern-0pt} {\sigma_{2} }}} {\sigma_{3} }}} \right. \kern-0pt} {\sigma_{3} }}$: The principal stresses (MPa)
$\sigma_{e}$: The von Mises failure criteria (MPa)
$\sigma_{p}$: The plastic stress (MPa)
$\Delta H$: The enthalpy change (J)
