# Supplementary material for: Experimental analysis and safety assessment of thermal runaway behavior in lithium iron phosphate batteries under mechanical abuse
Source: Sci Rep. 2024 Apr 15;14:8673. doi: 10.1038/s41598-024-58891-1 (PMC11018818; doi:10.1038/s41598-024-58891-1)
Supplement: Supplementary file 1 — Supplementary Information. [file 41598_2024_58891_MOESM1_ESM.docx]

**Supplemental Information**

**Experimental Analysis and Safety Assessment of Thermal Runaway Behavior in Lithium Iron Phosphate Batteries under Mechanical Abuse**

Zhixiong Chai, Junqiu Li, Ziming Liu， Zhengnan Liu& Xin Jin

# Materials and Methods

Figure S1 illustrates the physical appearance of the battery. Figure S1(a) displays the battery's three-dimensional structure and exterior view, while Figure S1(b) shows the CT image of the battery. The images show that the battery consists of two similar core components connected in series, enclosed by an outer steel shell structure.


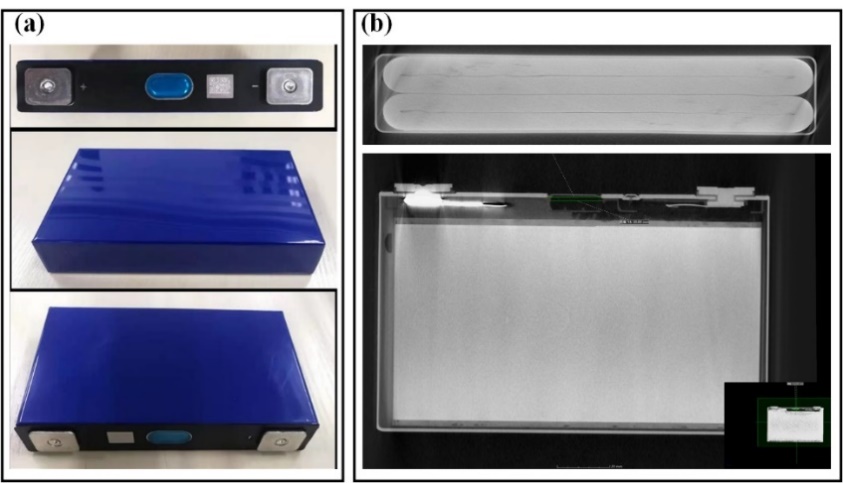


Figure S1 LFP battery (a)Three-dimensional battery geometry；(b)CT image

Figure S2 depicts the voltage and current curves of an LFP32 battery cycled three times. Initially, the current was discharged at 0.5C to 2.5V, followed by three cycling conditions. The cycling conditions comprised a "CC-CV" charge procedure, two rested procedures, and a CC discharge procedure. During the "CC-CV" charge procedure, the cells were charged to 3.65V using a constant current of 16A (CC, 0.5C), followed by a constant voltage (CV, 3.65V) charging process until the charge current decreased to 1.6A (0.05C). The resting procedures involved a 2-hour resting period. Finally, during the CC discharge procedure, the cells were discharged to 2.5V with a constant current of 16A (CC, 0.5C).


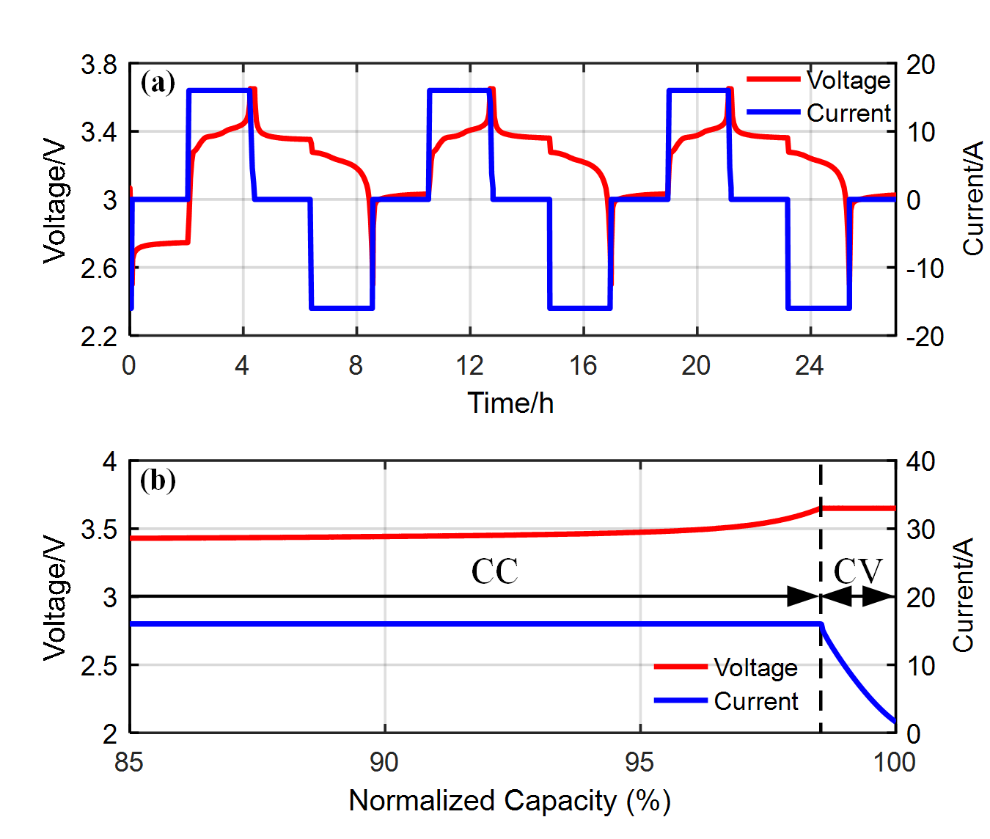


Figure S2 (a) Battery cycling conditions； (b) CC-CV charging process

# Results and discussions

Figure S3 shows the force-displacement data collected by the extrusion machine.


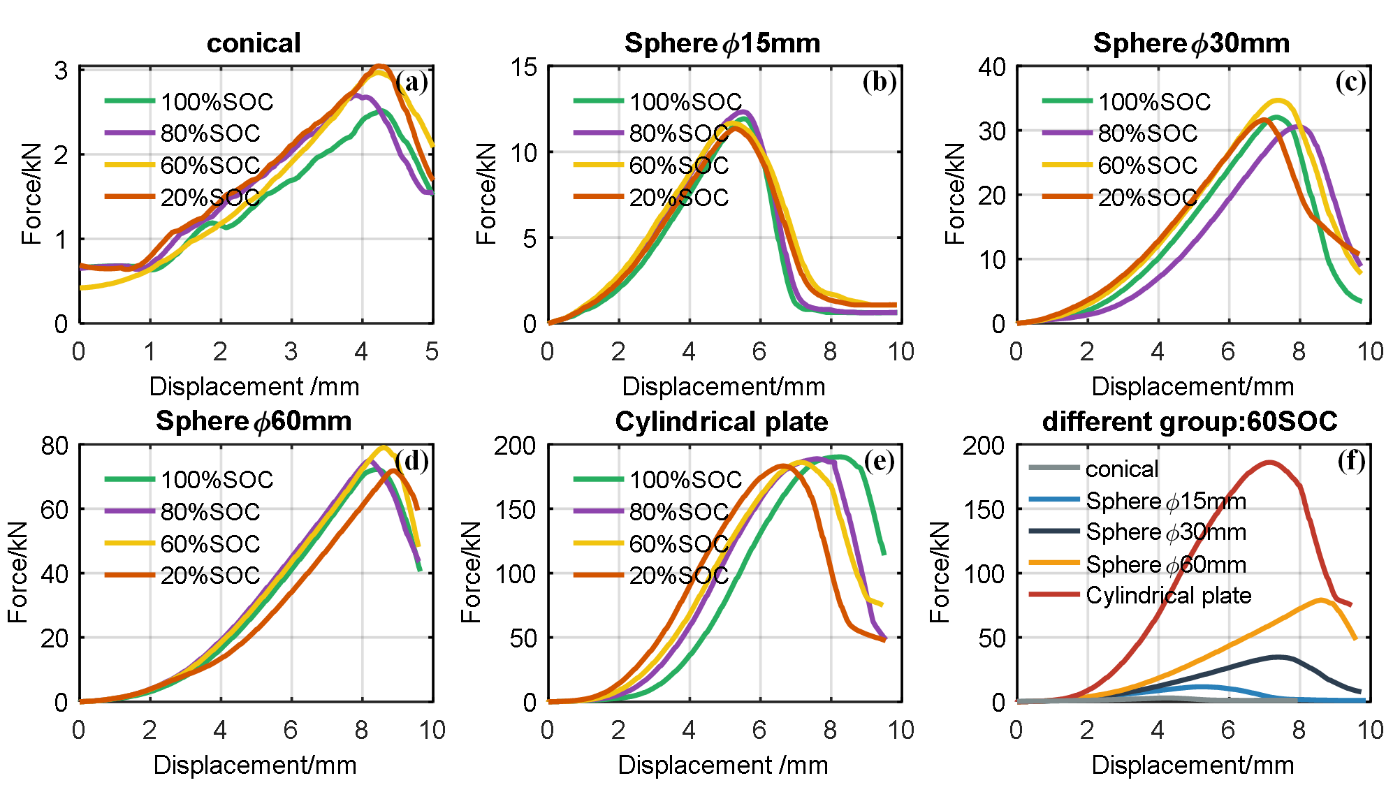


Figure S3 Mechanical response under different SOC with different punches. (a)~(e) Mechanical response under different SOCs；(f) Mechanical response of different groups of 60SOC batteries

Figure S4 shows the voltage-time data collected by the data acquisition instrument (DAQ).


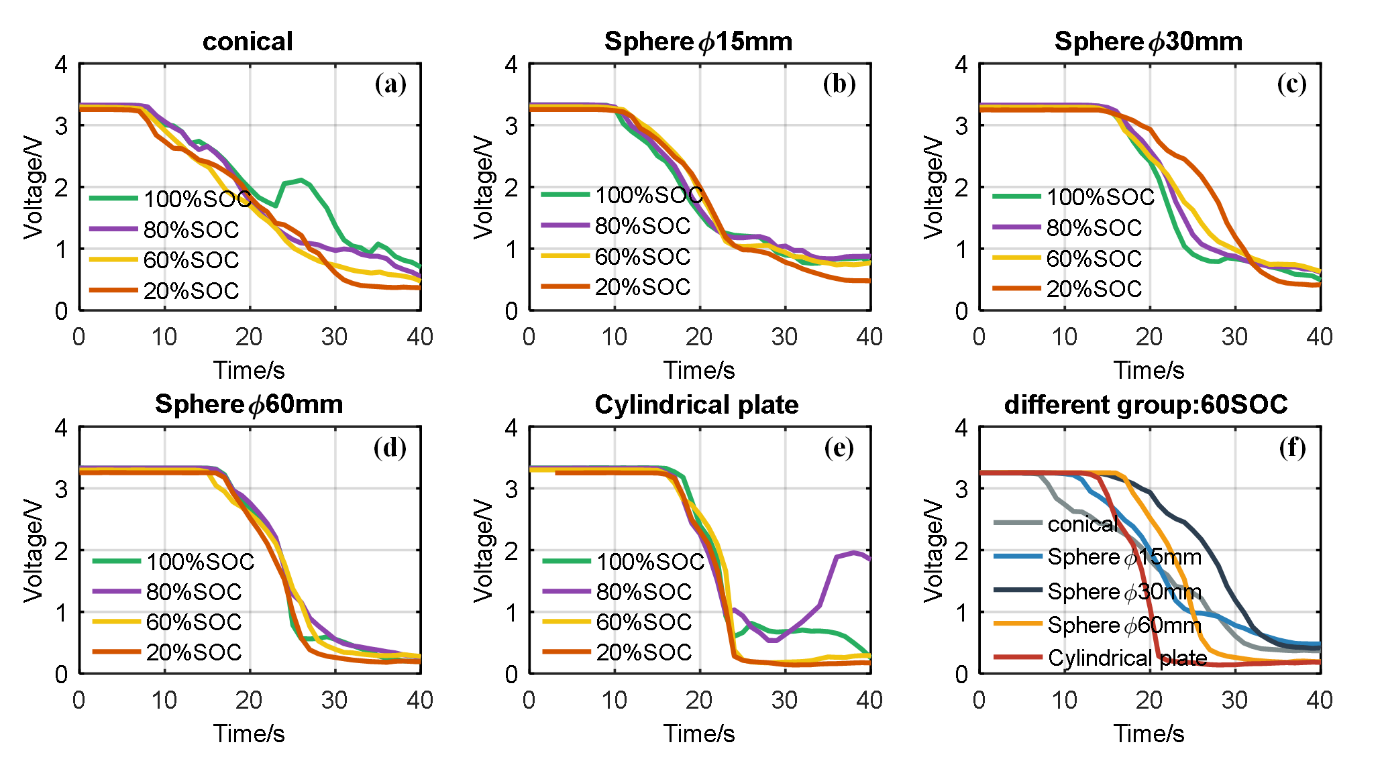


Figure S4 Electrical response under different SOC with different punches. (a)~(e) Electrical response under different SOCs；(f) Electrical response of different groups of 60SOC batteries

Figure S5 shows the measured temperature-time curve and MTD data collected by DAQ.


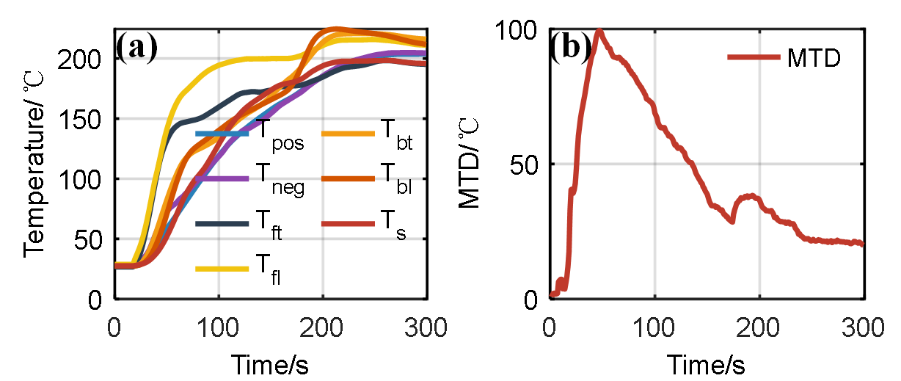


Figure S5 Battery temperature under mechanical abuse. (a) Battery temperature at different positions; (b) Maximum battery temperature difference.

Figure S6 shows the measured temperature-time data collected by DAQ.


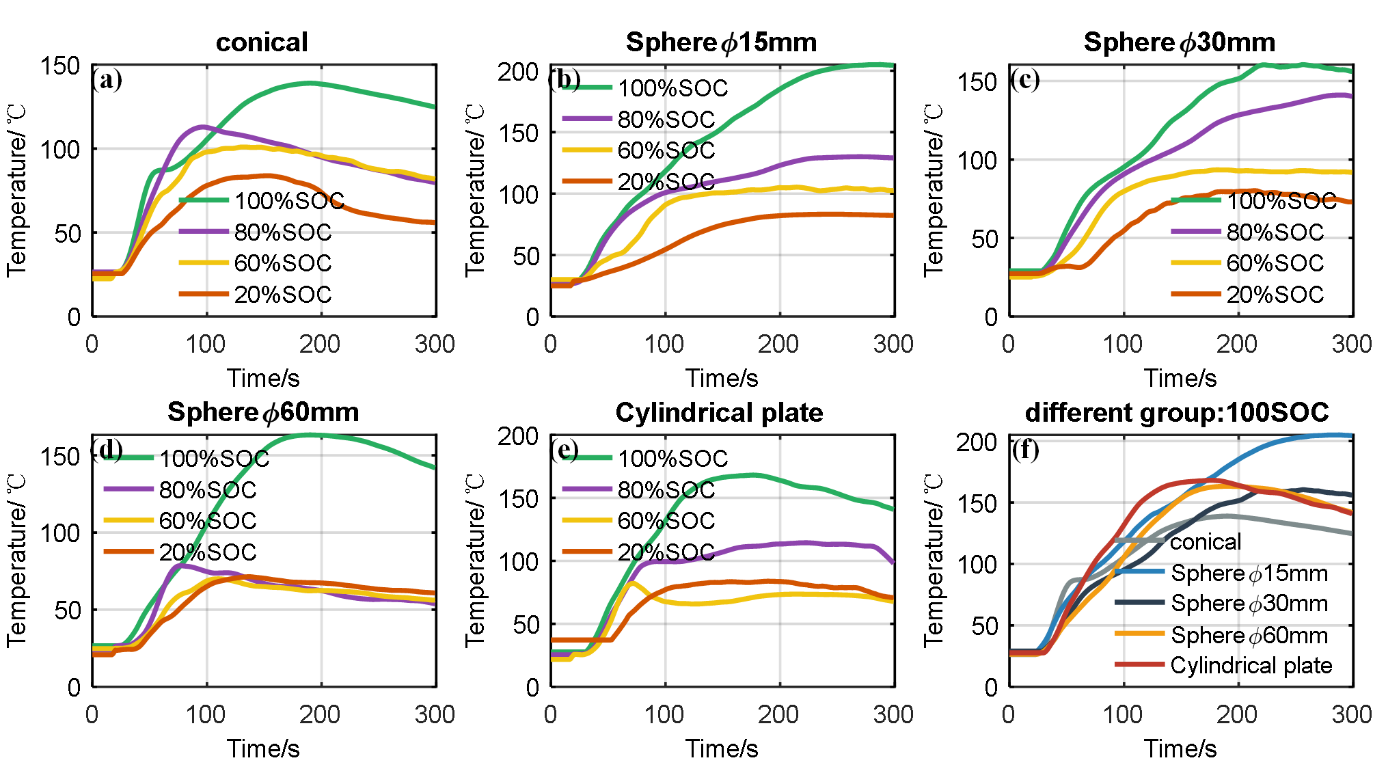


Figure S6 Thermal response under different SOC with different punches. (a)~(e) Thermal response under different SOCs；(f) Thermal response of different groups of 60SOC batteries.

# Safety assessment of thermal runaway behavior

Figure S7(a) shows the battery core extracted from the battery, and Figure S7(b) shows the stress-strain curve of the battery core obtained from the flat plate extrusion experiment fitted using the isotropic model. The fitted equation is:

$$\sigma=8135\varepsilon^{2.7}+0.8$$

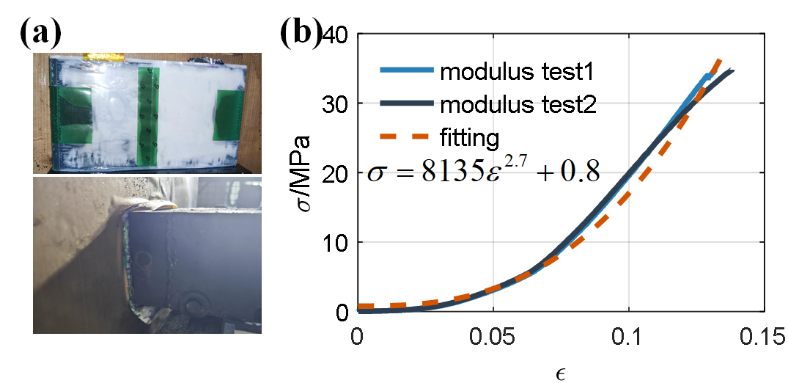


Figure S7 Jell roll module test. (a) Jell roll extrusion test; (b) Strain curve calculation and fitting.

Thermal images of two typical experiments are shown in Figure S8, taking the 30mm spherical punch with 100% SOC battery experiment as an example, where the temperatures of the battery, punch, and block reach 150°C, 85°C, and 69°C, respectively, aligning with the temperature-time curve in Figure S6.


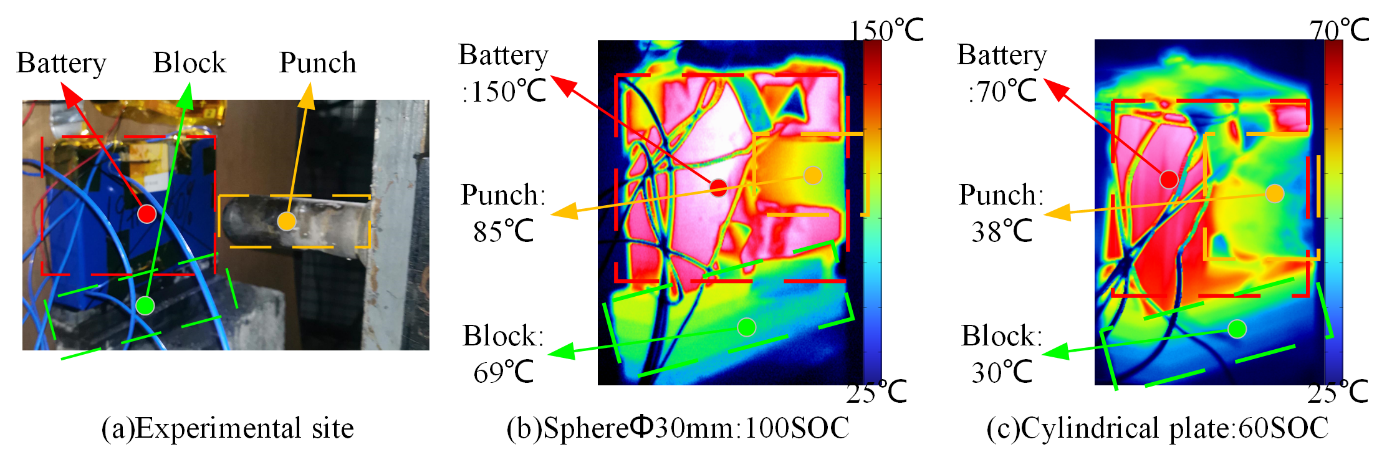


Figure S8 (a)Experimental site; (b)thermal imaging after 200s 100SOC battery under sphere ɸ30mm punch；(b) thermal imaging after 200s 60SOC battery under cylindrical plate punch

Comparing and analyzing the appearance of the battery remnants after the experiment, taking the conical condition with the lowest enthalpy change as an example, Figure S9 shows the appearance after the experiment.


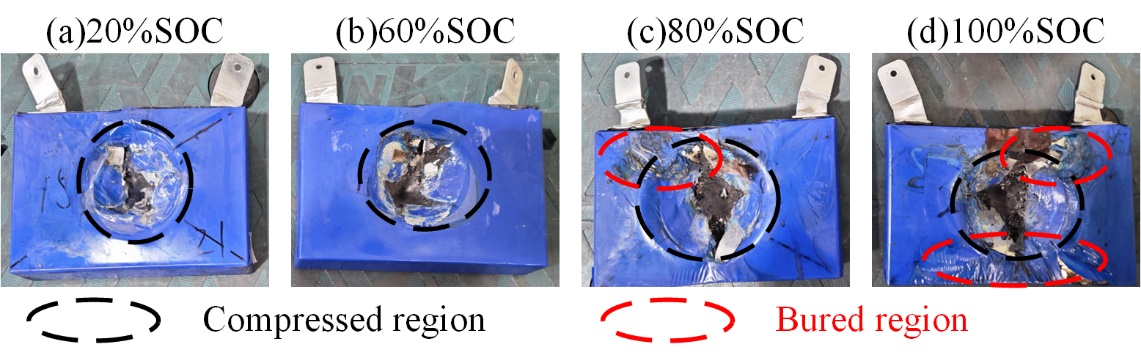


Figure S9 The appearance of the battery after the experiment: conical punch
